# Assessing the Safety of a Replacement Chemical: Nongenomic Activity of Bisphenol S

**DOI:** 10.1289/ehp.121-a97

**Published:** 2013-03-01

**Authors:** Julia R. Barrett

**Affiliations:** Julia R. Barrett, MS, ELS, a Madison, WI–based science writer and editor, has written for *EHP* since 1996. She is a member of the National Association of Science Writers and the Board of Editors in the Life Sciences.

Suspected links between bisphenol A (BPA) and adverse human health effects have spurred a search for replacement chemicals for use in common applications such as polycarbonate plastics, food can linings, and thermal papers. Bisphenol S (BPS) has been adopted for some uses. A new study now shows that low doses of BPS can disrupt nongenomic signaling pathways in cultured pituitary cells [*EHP 121(3):352–358; Viñas and Watson*].

Nearly 93% of U.S. residents over age 6 carry measurable amounts of BPA in their bodies. The compound is suspected to contribute to the development of diseases such as diabetes, asthma, and cancer, and animal studies have shown it adversely affects reproduction. In contrast to genomic regulation, which involves receptors in the cell nucleus, nongenomic regulation involves receptors in the cell membrane. Since BPA can interact with cell-membrane estrogen receptors, researchers explored whether structurally similar BPS might likewise influence nongenomic pathways.

Treatments included various low doses of BPS alone and paired with estradiol, an endogenous estrogen. Measured responses included activation of ERK and JNK, mitogen-activated protein kinases involved in cell growth, proliferation, and apoptosis (programmed cell death); activation of caspases 8 and 9, enzymes that also are involved in apoptosis; and release of prolactin, a hormone that helps regulate hundreds of biological functions, including metabolism, reproduction, and lactation.

**Figure:**
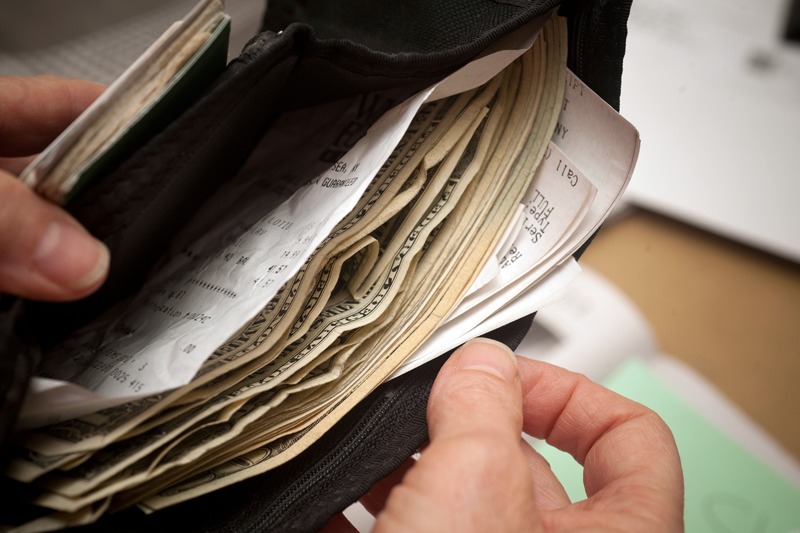
BPS has been adopted as a replacement for BPA in applications such as thermal receipt paper. © Richard Levine/Alamy

BPS appeared to interact predominantly with the cell-membrane estrogen receptor ER〈. Estradiol and BPS each increased cell proliferation, but following exposure to both compounds there were fewer cells than in control cultures. This can result from decreased proliferation or increased cell death, which is why the investigators also looked at caspase 8 and caspase 9. The former was activated by BPS alone and when combined with estradiol, but caspase 9 was less affected, consistent with an increase in apoptosis.

The lowest concentrations of BPS triggered the strongest ERK response; however, ERK activation was not as strong in the presence of both compounds. BPS alone did not activate JNK, but in combination with estradiol it increased JNK activation more than the estrogen alone. The quality and timing of ERK and JNK responses to activation by estradiol were often altered by BPS. By itself, BPS did not affect prolactin secretion, but it significantly suppressed estradiol-induced secretion at most concentrations.

Based on these results, low concentrations of BPS appear to affect nongenomic signaling in estrogen-responsive cells, with potential consequences for cell function. The results emphasize the need to screen compounds for estrogenic activity prior to their use in manufacturing.

